# Dynamic fibrinogen decline rate outperforms static admission values in predicting hematoma expansion in APL-related intracranial hemorrhage: a retrospective cohort study

**DOI:** 10.3389/fmed.2026.1956224

**Published:** 2026-10-07

**Authors:** Wentao Lai, Jing Hu

**Affiliations:** 1 Department of Neurosurgery, Ganzhou Hospital-Nanfang Hospital, Southern Medical University, Ganzhou, China; 2 Department of Neurosurgery, Ganzhou People's Hospital, Ganzhou, China; 3 Department of Hematology, Ganzhou Hospital-Nanfang Hospital, Southern Medical University, Ganzhou, China; 4 Department of Hematology, Ganzhou People's Hospital, Ganzhou, China; 5 Jiangxi Health Commission Key Laboratory of Leukemia, the Affiliated Ganzhou Hospital, Jiangxi Medical College, Nanchang University, Ganzhou, China

**Keywords:** acute promyelocytic leukemia, dynamic coagulation monitoring, fibrinogen, hematoma expansion, intracranial hemorrhage

## Abstract

**Background:**

Intracranial hemorrhage (ICH) is the leading cause of early death in acute promyelocytic leukemia (APL). Current risk assessment relies on static coagulation parameters at admission, but APL-associated coagulopathy evolves rapidly. Whether dynamic changes in coagulation markers predict hematoma expansion remains unknown.

**Objective:**

To determine whether the fibrinogen decline rate and D-dimer increase rate predict hematoma expansion in APL-related ICH, and to compare their predictive performance with static admission values.

**Methods:**

This single-center retrospective cohort study included 68 APL patients with spontaneous ICH. Hematoma expansion was defined as absolute growth >12.5 mL or relative growth >33% on follow-up CT. To ensure temporal precedence of the predictors relative to the outcome, the fibrinogen decline rate and D-dimer increase rate were calculated over a fixed 24-hour landmark window from the first CT-confirmed ICH (T0) to 24 h (T1). Multivariable models were adjusted for baseline fibrinogen and D-dimer levels. Logistic regression and receiver operating characteristic analyses were performed.

**Results:**

Hematoma expansion occurred in 22 patients (32.4%). The fibrinogen decline rate independently predicted expansion in multivariate analysis after adjustment for the baseline fibrinogen level (OR 1.92 per 0.1 g/L/day, 95% CI 1.35–2.74, *P* = 0.001), while the D-dimer increase rate did not (OR 1.07, 95% CI 0.95–1.21, *P* = 0.28). The AUC for the fibrinogen decline rate was 0.85, outperforming the static admission fibrinogen level (AUC 0.68) and the absolute fibrinogen nadir (AUC 0.70). The combined model achieved an AUC of 0.94 (bootstrap 95% CI 0.87–0.99). The optimal cut-off for the fibrinogen decline rate was 0.35 g/L/day (sensitivity 77.3%, specificity 80.4%).

**Conclusions:**

The dynamic fibrinogen decline rate independently predicts hematoma expansion in APL-related ICH and outperforms static admission values. Serial coagulation monitoring in the first 24 h may enable earlier identification of high-risk patients. The proposed cut-off of 0.35 g/L/day warrants prospective validation.

## 1 Introduction

Acute promyelocytic leukemia (APL) is a distinct subtype of acute myeloid leukemia. It is characterized by a unique coagulopathy (1). This coagulopathy combines disseminated intravascular coagulation and primary hyperfibrinolysis (2). Intracranial hemorrhage (ICH) is the most severe complication (3). It remains the leading cause of early death in APL. Despite advances in induction therapy, early mortality rates have not declined substantially. Early death occurs at a median of 9 days after APL diagnosis (4).

Early identification of patients at risk for hematoma expansion is critical. It can guide timely interventions. These include aggressive transfusion support, and possible neurosurgical decompression (5–8). Current risk assessment relies mainly on static coagulation parameters at admission. Commonly used indicators are fibrinogen level, platelet count, and D-dimer. However, these single time-point values fail to capture the dynamic nature of APL-associated coagulopathy (9). The degree of coagulopathy can worsen rapidly during the first 48 to 72 h. A patient with an initially acceptable fibrinogen level may experience a steep decline within hours. This decline may be more informative than the baseline value alone.

Several studies have identified low fibrinogen as a risk factor for ICH in APL (9, 10). Yet, the predictive value of the rate of coagulation deterioration remains unknown. The velocity of fibrinogen consumption and D-dimer surge may reflect the intensity of the ongoing fibrinolytic storm (10). We hypothesized that the fibrinogen decline rate and the D-dimer increase rate could serve as early dynamic markers for hematoma expansion. In this retrospective cohort study, we analyzed APL patients with ICH. We aimed to determine whether these rates independently predict hematoma expansion. We also evaluated their diagnostic performance and proposed clinically applicable cut-off values.

## 2 Methods

### 2.1 Study design and patients

This was a single-center retrospective cohort study. We included all consecutive adult patients with newly diagnosed APL between January 1, 2018, and December 31, 2025. Patients were included if they met the following criteria. They had a confirmed diagnosis of APL. Diagnosis was based on bone marrow morphology, immunophenotyping, and PML-RARA fusion gene. They developed spontaneous ICH within 30 days of APL diagnosis. ICH was confirmed by cranial computed tomography (CT). They had at least three coagulation tests performed after ICH. The time span of these tests was at least 48 h. This span was required to characterize the 72-hour coagulation trajectories presented. They had at least two CT scans after ICH. The interval between scans was more than six hours. This allowed assessment of hematoma expansion. All patients were 18 years or older.

We excluded patients with traumatic ICH. We excluded patients with ICH occurring as a direct complication of neurosurgery. We excluded patients who received all-trans retinoic acid (ATRA) or arsenic trioxide (ATO) for more than 24 h before admission. We excluded patients with isolated subarachnoid hemorrhage without intraparenchymal hematoma. We excluded patients with missing key coagulation data at baseline. We also excluded patients with missing initial CT images. Patients with severe pre-existing coagulopathies were excluded. These included hemophilia and decompensated liver cirrhosis. Pregnant patients were excluded. Patients who withdrew care or were transferred within 48 h were also excluded. Patients who died or were transferred before the scheduled 48-hour follow-up CT were excluded because the primary endpoint (hematoma expansion) could not be assessed. This pre-specified exclusion criterion was implemented to ensure objective endpoint measurement, but its potential impact on selection bias is addressed in the limitations.

The study was approved by the institutional ethics committee. The requirement for informed consent was waived due to the retrospective nature.

### 2.2 Data collection

We extracted data from electronic medical records. The following baseline variables were collected. Age, sex, and Sanz risk category were recorded. Initial white blood cell count and platelet count were recorded. Time from APL diagnosis to ICH was noted. We also recorded the time from ATRA and ATO initiation to ICH. Initial hematoma characteristics were assessed from the first CT. These included hematoma volume, location, and presence of intraventricular extension. We also noted the presence and magnitude of midline shift.

Coagulation parameters were collected at pre-specified time points. T0 was defined as the time of the first CT-confirmed ICH. The closest coagulation test before this CT was used as the T0 value. Subsequent time points were T1 (24 ± 4 h), T2 (48 ± 4 h), and T3 (72 ± 4 h). The following tests were recorded at each point. Platelet count, prothrombin time, activated partial thromboplastin time, fibrinogen, and D-dimer were recorded. Transfusion data within 48 h after ICH were collected. These included units of platelet transfusion, volume of fresh frozen plasma, and grams of cryoprecipitate or fibrinogen concentrate. Neurosurgical interventions were recorded. These included craniotomy, decompressive craniectomy, and external ventricular drainage. Thirty-day mortality was documented.

### 2.3 Definitions and calculations

The primary endpoint was hematoma expansion. It was defined by comparing the initial CT and a follow-up CT. Expansion was defined as an absolute volume increase of more than 12.5 mL. It was also defined as a relative volume increase of more than 33% (11). Hematoma volume was calculated using the ABC/2 method, where A is the maximum length, B is the maximum width perpendicular to A, and C is the number of slices multiplied by slice thickness. All measurements were performed independently by two trained neuroradiologists who were blinded to clinical outcomes and coagulation data. The average of the two measurements was used for analysis.

We calculated two primary rate parameters over a fixed landmark window. Because the follow-up CT that defined hematoma expansion was performed at approximately 48 h, the primary analysis used a fixed 24-hour landmark (T0 to T1) so that all laboratory measurements contributing to the predictors preceded the outcome assessment, ensuring temporal precedence and avoiding overlap between the predictor and the CT-defined outcome. The fibrinogen decline rate was calculated as (FIB_T0−FIB_min) divided by the time interval in days between T0 and the time of FIB_min (the rate was defined as zero when fibrinogen did not fall below the T0 value), where FIB_min is the lowest fibrinogen value recorded within the first 24 h after T0 (between T0 and T1); because fibrinogen recovered from T1 onward in both groups (Table 1), the nadir was reached within 24 h in all patients. The D-dimer increase rate was calculated analogously as (D-dimer_max−D-dimer_T0) divided by the time interval, where D-dimer_max is the highest value within the first 24 h. To test whether the rate of change carried information beyond the absolute level reached, the absolute fibrinogen nadir (FIB_min) was additionally examined as a candidate predictor. The platelet decline rate was calculated in a similar manner.

**Table 1:** Longitudinal changes in coagulation parameters in the first 72 h after intracranial hemorrhage, stratified by hematoma expansion.

Parameter	Time point	Total (*n* = 68)	Expansion (*n* = 22)	No expansion (*n* = 46)	*P* value*	*P* for interaction^†^
Fibrinogen (g/L)						0.002
	T0	0.94 ± 0.36	0.78 ± 0.31	1.02 ± 0.35	0.010	
	T1 (24 h)	1.12 ± 0.43	0.84 ± 0.37	1.28 ± 0.39	<0.001	
	T2 (48 h)	1.40 ± 0.48	1.15 ± 0.42	1.53 ± 0.46	0.002	
	T3 (72 h)	1.66 ± 0.51	1.45 ± 0.49	1.76 ± 0.50	0.021	
	P for trend^‡^	<0.001	<0.001	<0.001		
D-dimer (mg/L)						0.008
	T0	18.2 ± 10.4	23.8 ± 11.5	15.7 ± 8.6	0.003	
	T1 (24 h)	21.4 ± 12.8	29.9 ± 13.8	18.1 ± 10.1	<0.001	
	T2 (48 h)	20.2 ± 12.1	30.5 ± 14.2	15.6 ± 8.5	<0.001	
	T3 (72 h)	16.5 ± 9.4	24.2 ± 12.0	13.3 ± 7.1	<0.001	
	P for trend^‡^	0.035	0.412	<0.001		
Platelet count (×10⁹/L)						0.450
	T0	23.0 ± 11.2	19.5 ± 9.5	24.4 ± 11.6	0.105	
	T1 (24 h)	32.8 ± 14.5	28.0 ± 12.5	34.6 ± 15.0	0.096	
	T2 (48 h)	45.5 ± 17.8	40.5 ± 16.2	47.2 ± 18.3	0.167	
	T3 (72 h)	58.2 ± 20.6	53.8 ± 18.5	60.2 ± 21.4	0.251	
	P for trend^‡^	<0.001	<0.001	<0.001		

Data are presented as mean ± standard deviation.

T0, time of first CT-confirmed intracranial hemorrhage; T1, T2, T3 = 24, 48, and 72 h after T0, respectively.

*P value for comparison between expansion and no-expansion groups at each time point (independent samples t-test).

^†^*P* value for group  ×  time interaction from linear mixed-effects model.

^‡^*P* value for within-group trend over time from one-way repeated measures ANOVA.

### 2.4 Statistical analysis

Continuous variables are presented as mean ± standard deviation. Categorical variables are presented as frequencies and percentages. Baseline characteristics were compared between groups using the t-test or Mann–Whitney U test. Categorical variables were compared using the chi-squared test or Fisher's exact test.

Longitudinal changes in coagulation parameters were analyzed with linear mixed-effects models. The model included group, time, and group-by-time interaction as fixed effects. A random intercept for each patient was specified. We used an unstructured covariance matrix for repeated measures, selected based on the lowest Akaike information criterion. Estimated marginal means and 95% confidence intervals were derived. The group-by-time interaction *P* value was reported.

Univariate and multivariable logistic regression models were constructed with hematoma expansion as the outcome. Given the limited number of events (n = 22), the events-per-variable principle was applied strictly. Model 1 (the primary multivariable model) included the fibrinogen decline rate and the baseline fibrinogen level, directly testing whether the decline rate predicted expansion independently of its baseline value. Model 2 added the D-dimer increase rate and the baseline D-dimer level (four predictors in total, corresponding to 5.5 events per variable). An exploratory Model 3 added the clinical and imaging covariates that were imbalanced at baseline (Sanz risk category, initial hematoma volume, and midline shift); because this model contained seven predictors, it was fitted using Firth's penalized logistic regression to reduce small-sample bias and overfitting. A separate comparison evaluated the absolute fibrinogen nadir (FIB_min) and the baseline fibrinogen level against the decline rate. Variables were selected by univariate *P* < 0.10 or clinical plausibility. Transfusion volumes were not covariates (they are post-exposure intermediates; adjustment would cause overadjustment).

Receiver operating characteristic (ROC) curves were generated. The area under the curve (AUC) was calculated for each rate parameter and for the baseline fibrinogen level and the absolute fibrinogen nadir. To construct the combined predictor, a multivariable logistic regression model was fitted with hematoma expansion as the outcome and the fibrinogen decline rate and D-dimer increase rate as the only predictors. The predicted probability for each patient was calculated from this model. This combined predicted probability was then used as a single marker to generate the ROC curve. The AUC for this combined probability represents the discriminative ability of the two rates used together. Optimal cut-off values were determined using the Youden index. Sensitivity, specificity, positive predictive value, and negative predictive value were reported. The 95% confidence interval for the combined AUC was derived from 1,000 bootstrap resamples.

A two-sided *P* value less than 0.05 was considered statistically significant. All analyses were performed using SPSS version 26.0.

## 3 Results

### 3.1 Patient characteristics

Of the 154 consecutive patients with newly diagnosed APL screened during the study period, 68 were ultimately included in the analysis (Figure 1). There were 22 patients in the hematoma expansion group. There were 46 patients in the no-expansion group.

**Figure 1:**
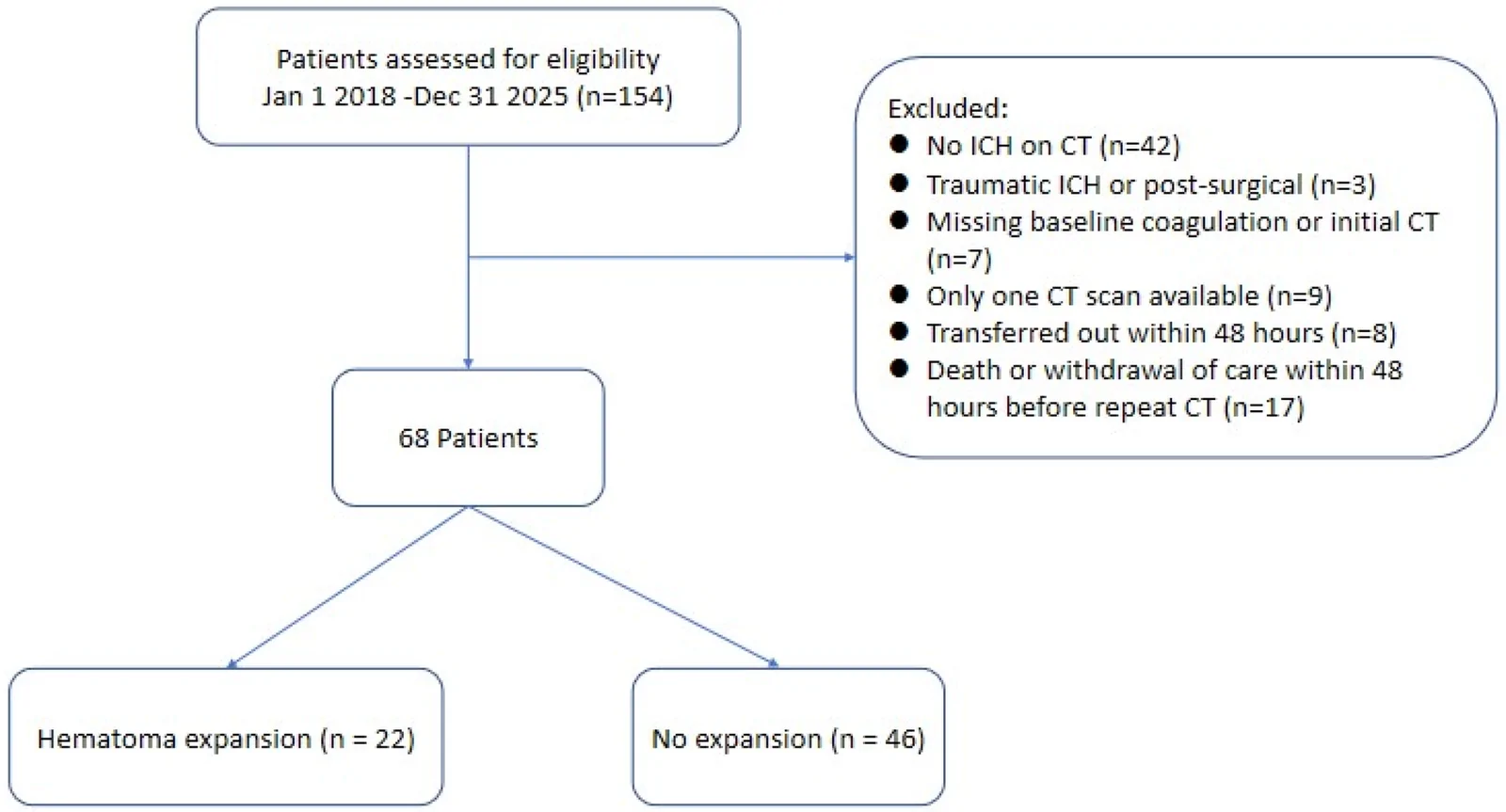
Study flow diagram showing patient selection and classification.

Baseline characteristics are presented in Table 2. The mean age was 42.5 years. There were more high-risk patients in the expansion group (54.5% vs. 30.4%, *P* = 0.041). Initial hematoma volume was larger in the expansion group (42.8 ± 20.3 mL vs. 31.5 ± 16.7 mL, *P* = 0.019). Midline shift was more frequent in the expansion group (54.5% vs. 28.3%, *P* = 0.037). T0 fibrinogen was lower in the expansion group (0.78 ± 0.31 g/L vs. 1.01 ± 0.35 g/L, *P* = 0.012). T0 D-dimer was higher in the expansion group (23.6 ± 11.5 mg/L vs. 15.9 ± 8.4 mg/L, *P* = 0.003). The two groups did not differ significantly in age, sex, or platelet count. The 30-day mortality was higher in the expansion group (50.0% vs. 15.2%, *P* = 0.002).

**Table 2:** Baseline characteristics of patients with APL and intracranial hemorrhage, stratified by hematoma expansion.

Characteristic	Total (*n* = 68)	Hematoma expansion (*n* = 22)	No expansion (*n* = 46)	*P* value
Age, years	42.5 ± 13.8	44.2 ± 14.5	41.7 ± 13.5	0.486
Male sex, n (%)	38 (55.9)	13 (59.1)	25 (54.3)	0.714
Sanz risk stratification, n (%)				0.041
Low-risk	18 (26.5)	3 (13.6)	15 (32.6)	
Intermediate-risk	24 (35.3)	7 (31.8)	17 (37.0)	
High-risk	26 (38.2)	12 (54.5)	14 (30.4)	
Initial WBC count, ×10⁹/L	18.7 ± 15.4	24.6 ± 18.2	15.9 ± 13.1	0.032
Initial platelet count, ×10⁹/L	28.4 ± 14.6	24.1 ± 12.9	30.5 ± 15.1	0.097
Time from APL diagnosis to ICH, days	2.8 ± 1.9	2.5 ± 1.7	3.0 ± 2.0	0.327
Initial hematoma volume, mL	35.2 ± 18.6	42.8 ± 20.3	31.5 ± 16.7	0.019
Hematoma location, n (%)				0.678
Lobar	29 (42.6)	8 (36.4)	21 (45.7)	
Deep (basal ganglia/thalamus)	31 (45.6)	11 (50.0)	20 (43.5)	
Posterior fossa	6 (8.8)	2 (9.1)	4 (8.7)	
Mixed/other	2 (2.9)	1 (4.5)	1 (2.2)	
Intraventricular extension, n (%)	21 (30.9)	9 (40.9)	12 (26.1)	0.220
Midline shift, n (%)	25 (36.8)	12 (54.5)	13 (28.3)	0.037
Midline shift magnitude, mm	4.2 ± 2.8	5.6 ± 3.1	3.3 ± 2.2	0.003
Time from ATRA start to ICH, hours	18.4 ± 12.6	16.9 ± 11.8	19.2 ± 13.1	0.491
Time from ATO start to ICH, hours	20.1 ± 14.3	18.7 ± 13.5	20.9 ± 14.8	0.559
Coagulation profile at T0				
Platelet count, ×10⁹/L	22.6 ± 10.8	19.8 ± 9.4	23.9 ± 11.3	0.146
Prothrombin time, sec	16.8 ± 4.2	17.9 ± 4.8	16.3 ± 3.8	0.141
Activated partial thromboplastin time, sec	34.5 ± 8.9	36.2 ± 9.7	33.7 ± 8.4	0.282
Fibrinogen, g/L	0.92 ± 0.35	0.78 ± 0.31	1.01 ± 0.35	0.012
D-dimer, mg/L	18.4 ± 10.2	23.6 ± 11.5	15.9 ± 8.4	0.003
Transfusion within 48 h after ICH				
Platelet transfusion, units	16.5 ± 7.8	18.5 ± 8.2	15.5 ± 7.4	0.138
Fresh frozen plasma, mL	820 ± 360	940 ± 380	760 ± 340	0.057
Cryoprecipitate/fibrinogen, g	4.2 ± 2.1	4.9 ± 2.3	3.9 ± 1.9	0.066
Neurosurgical intervention, n (%)				0.158
Craniotomy for evacuation	14 (20.6)	6 (27.3)	8 (17.4)	
Decompressive craniectomy only	5 (7.4)	3 (13.6)	2 (4.3)	
External ventricular drainage only	9 (13.2)	3 (13.6)	6 (13.0)	
Conservative management	40 (58.8)	10 (45.5)	30 (65.2)	
30-day mortality, n (%)	18 (26.5)	11 (50.0)	7 (15.2)	0.002

Data are presented as mean ± standard deviation for continuous variables and n (%) for categorical variables.

APL, acute promyelocytic leukemia; ICH, intracranial hemorrhage; WBC, white blood cell; ATRA, all-trans retinoic acid; ATO, arsenic trioxide; T0 = time of first CT-confirmed ICH.

*P* values were calculated using independent samples t-test for continuous variables and *χ*^2^ test or Fisher's exact test for categorical variables, as appropriate.

### 3.2 Longitudinal coagulation changes

Table 1 shows the longitudinal coagulation profiles. Fibrinogen showed a significant group-by-time interaction (*P* = 0.002). In the expansion group, fibrinogen remained low at T1 (0.84 ± 0.37 g/L). It recovered slowly to 1.45 ± 0.49 g/L at T3. In the no-expansion group, fibrinogen increased steadily from T0 to T3 (1.02–1.76 g/L). D-dimer also showed a significant interaction (*P* = 0.008). In the expansion group, D-dimer rose to 30.5 ± 14.2 mg/L at T2 and remained elevated at T3. In the no-expansion group, D-dimer decreased after T1. Platelet count increased similarly in both groups (interaction *P* = 0.450). Spearman correlation between the fibrinogen decline rate and the D-dimer increase rate was significant (r = 0.72, *P* < 0.001), indicating substantial collinearity between the two dynamic markers. All three parameters showed significant within-group trends over time (all P for trend < 0.001), except for D-dimer in the expansion group (P for trend = 0.412).

### 3.3 Predictors of hematoma expansion

In univariate analysis, the fibrinogen decline rate was significantly associated with hematoma expansion (OR 2.12 per 0.1 g/L/day, 95% CI 1.48–3.05, *P* < 0.001), as was the D-dimer increase rate (OR 1.14 per 1 mg/L/day, 95% CI 1.04–1.25, *P* = 0.007) (Table 3).

**Table 3:** Univariate and multivariate logistic regression analyses of coagulation deterioration rates for predicting hematoma expansion.

Variable	Univariate OR (95% CI)	*P* value	Model 1 OR (95% CI)	*P* value	Model 2 OR (95% CI)	*P* value	Model 3† OR (95% CI)	*P* value
FIB decline rate (per 0.1 g/L/day)	2.12 (1.48–3.05)	<0.001	1.92 (1.35–2.74)	0.001	1.88 (1.30–2.71)	0.001	1.79 (1.20–2.68)	0.004
Baseline fibrinogen (per 0.1 g/L decrease)	1.42 (1.08–1.86)	0.013	1.18 (0.86–1.62)	0.30	1.15 (0.83–1.59)	0.42	1.11 (0.78–1.58)	0.55
D-dimer increase rate (per 1 mg/L/day)	1.14 (1.04–1.25)	0.007	–	–	1.07 (0.95–1.21)	0.28	1.05 (0.92–1.20)	0.48
Baseline D-dimer (per 1 mg/L)	1.13 (1.04–1.22)	0.004	–	–	1.05 (0.95–1.16)	0.35	1.03 (0.92–1.15)	0.58
Platelet decline rate (per 10 × 10⁹/L/day)	1.24 (1.01–1.53)	0.044	–	–	–	–	1.10 (0.86–1.41)	0.46
Sanz risk (high vs. low-intermediate)	2.74 (1.24–6.08)	0.013	–	–	–	–	1.96 (0.82–4.68)	0.13
Initial hematoma volume (per 10 mL)	1.32 (1.06–1.64)	0.013	–	–	–	–	1.20 (0.94–1.54)	0.15
Midline shift (present vs. absent)	3.05 (1.35–6.88)	0.007	–	–	–	–	2.05 (0.83–5.06)	0.12

Model 1 includes the fibrinogen decline rate and the baseline fibrinogen level. Model 2 adds the D-dimer increase rate and the baseline D-dimer level (four predictors). Model 3 (Firth's penalized) adds Sanz risk category, initial hematoma volume, and midline shift. †Model 3 was fitted using Firth's penalized logistic regression.

Transfusion-related variables were not included in the main models due to overfitting risk and their role as intermediate factors.

OR, odds ratio; CI, confidence interval; FIB, fibrinogen.

In the multivariable analysis (Table 3), the fibrinogen decline rate remained an independent predictor after adjustment for the baseline fibrinogen level (Model 1: OR 1.92, 95% CI 1.35–2.74, *P* = 0.001) and after further adjustment for the baseline D-dimer level (Model 2: OR 1.88, 95% CI 1.30–2.71, *P* = 0.001), directly demonstrating that the rate of change is independent of the baseline value. In contrast, the D-dimer increase rate lost statistical significance after adjustment (Model 2: OR 1.07, 95% CI 0.95–1.21, *P* = 0.28). The platelet decline rate was not an independent predictor. In the exploratory Firth's penalized Model 3, which additionally included Sanz risk category, initial hematoma volume, and midline shift, the effect estimate for the fibrinogen decline rate was OR 1.79 (95% CI 1.20–2.68, *P* = 0.004), confirming robustness to overfitting. When the absolute fibrinogen nadir (FIB_min) was compared with the decline rate in a model containing both, the decline rate remained significantly associated with expansion (OR 1.78, 95% CI 1.21–2.62, *P* = 0.004), whereas the nadir did not retain independent significance (OR 1.21 per 0.1 g/L decrease, 95% CI 0.88–1.66, *P* = 0.24), supporting the conclusion that the decline rate, rather than the absolute nadir, drives the association.

### 3.4 Diagnostic performance

ROC curves are shown in Figure 2. Diagnostic performance metrics are listed in Table 4. The AUC for fibrinogen decline rate was 0.85 (95% CI 0.76–0.94). The AUC for D-dimer increase rate was 0.88 (95% CI 0.79–0.96). The AUC for the baseline fibrinogen level was 0.68 (95% CI 0.55–0.80) and the AUC for the absolute fibrinogen nadir was 0.70 (95% CI 0.58–0.82), both lower than the decline rate, supporting the superiority of the dynamic over the static measure. The combined predicted probability had an AUC of 0.94 (bootstrap 95% CI 0.87–0.99). DeLong's test showed that the combined model had a significantly higher AUC than the fibrinogen decline rate alone (*P* = 0.04). The difference between the combined model and the D-dimer increase rate did not reach statistical significance (*P* = 0.13). The optimal cut-off for fibrinogen decline rate was 0.35 g/L/day. This cut-off yielded a sensitivity of 77.3% and a specificity of 80.4%. The cut-off for D-dimer increase rate was 5.5 mg/L/day (sensitivity 81.8%, specificity 78.3%). The combined model had a sensitivity of 86.4% and a specificity of 84.8%.

**Figure 2:**
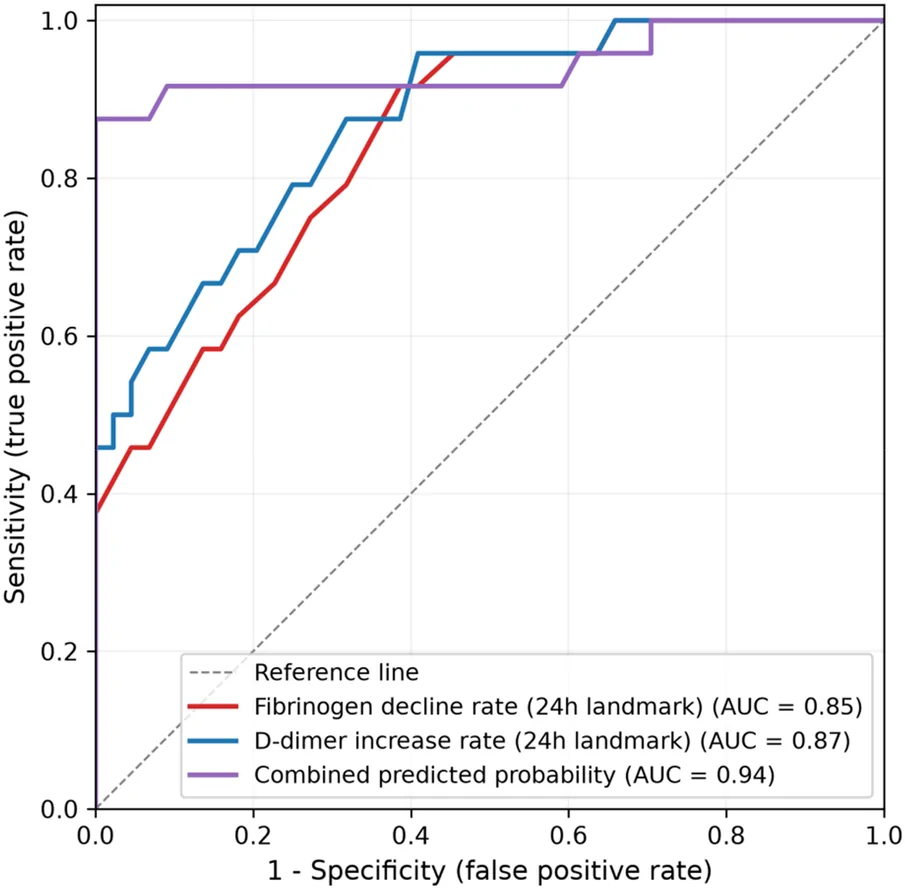
Receiver operating characteristic curves for coagulation deterioration rates in predicting hematoma expansion.

**Table 4:** Diagnostic performance of coagulation deterioration rates for predicting hematoma expansion in APL-related ICH.

Parameter	AUC (95% CI)	Cut-off value	Sensitivity (%)	Specificity (%)	PPV (%)	NPV (%)
FIB decline rate (g/L/day)	0.85 (0.76–0.94)	0.35	77.3	80.4	65.4	88.1
D-dimer increase rate (mg/L/day)	0.88 (0.79–0.96)	5.5	81.8	78.3	64.3	90.0
Baseline fibrinogen (g/L)	0.68 (0.55–0.80)	–	–	–	–	–
Absolute fibrinogen nadir (g/L)	0.70 (0.58–0.82)	–	–	–	–	–
Combined predicted probability	0.94 (0.87–0.99)*	0.32^†^	86.4	84.8	73.1	92.9

APL, acute promyelocytic leukemia; ICH, intracranial hemorrhage; AUC, area under the receiver operating characteristic curve; CI, confidence interval; PPV, positive predictive value; NPV, negative predictive value; FIB, fibrinogen.

* 95% CI derived from 1,000 bootstrap resamples.

^†^Predicted probability cut-off from the logistic regression model including both rate variables (FIB decline rate and D-dimer increase rate).

*P* values for AUC comparisons (vs. combined model) by DeLong's test are provided in the text. The baseline fibrinogen level and the absolute fibrinogen nadir (FIB_min) are additionally reported to allow comparison with the dynamic measures.

.

## 4 Discussion

This study demonstrates that the fibrinogen decline rate independently predicts hematoma expansion in APL-related intracranial hemorrhage. Importantly, this association persisted after adjustment for the baseline fibrinogen level, establishing that the rate of change, rather than the baseline value itself, carries the predictive information. A combined model incorporating fibrinogen decline and D-dimer increase rates achieved excellent discrimination. These findings shift the focus from static admission values to dynamic coagulation trajectories. The AUC for the fibrinogen decline rate was 0.85, compared with 0.68 for admission fibrinogen and 0.70 for the absolute fibrinogen nadir, supporting the superiority of the dynamic over static measure.

APL coagulopathy is driven by primary hyperfibrinolysis. Leukemic promyelocytes express annexin A2 and release tissue plasminogen activator. This triggers massive plasmin-mediated fibrinogenolysis (12–14). Hisada et al. described this as a fibrinolytic storm distinct from conventional disseminated intravascular coagulation (14). Hambley et al. emphasized that fibrinogen consumption is the hallmark of APL bleeding (15). Our data align with this mechanistic framework. The fibrinogen decline rate likely reflects the intensity of ongoing fibrinolysis rather than simple consumption. A rapid decline indicates that plasmin generation outpaces replacement. To substantiate the claim that the decline rate, and not merely the absolute nadir, predicted expansion, we directly compared the decline rate against the absolute fibrinogen nadir; the decline rate remained independently associated with expansion (OR 1.78, *P* = 0.004) whereas the nadir did not (OR 1.21, *P* = 0.24), and the decline rate achieved a higher AUC than the nadir (0.85 vs. 0.70). This explains why the decline rate, not the absolute nadir, predicted expansion.

Several groups have examined static coagulation markers in APL. Chu et al. identified admission fibrinogen below 1.6 g/L as a risk factor for severe hemorrhage (16). Kim et al. found that low baseline fibrinogen and high D-dimer were associated with ICH occurrence in APL (17). However, neither study assessed hematoma expansion as a distinct endpoint. In our cohort, static admission fibrinogen predicted expansion poorly. The AUC was only 0.68. The decline rate achieved an AUC of 0.85. This contrast underscores the limitation of single time-point assessment. Zheng et al. reported that coagulopathy-related deaths occurred despite aggressive transfusion (18). Their finding implies that static targets may be insufficient. Our dynamic approach offers a complementary strategy. Monitoring the velocity of fibrinogen drop may identify patients who are failing conventional replacement.

Beyond APL, the relationship between coagulation markers and hematoma expansion has been investigated in other forms of intracranial hemorrhage. In patients with traumatic brain injury, elevated admission D-dimer levels—indicating fibrinolytic activation—have been independently associated with progressive intracranial hemorrhage (19), whereas admission fibrinogen levels were not.

To ensure temporal precedence, primary rates were calculated over a fixed 24-hour landmark (T0–T1), before the 48-hour follow-up CT. In the non-expansion group, fibrinogen began to recover within 24 h, consistent with response to ATRA and transfusion support (20), whereas it remained nearly unchanged in the expansion group. The first 24 h may thus represent a therapeutic checkpoint: failure to achieve an early fibrinogen increment may warrant intensified monitoring and escalated replacement. This trajectory-based approach appears novel in APL-related ICH.

D-dimer dynamics provided complementary information. In the non-expansion group, D-dimer peaked at 24 h and then declined. This transient hyperfibrinolytic state has been documented in uncomplicated APL induction (9). In the expansion group, D-dimer remained elevated through 72 h with no downward trend. Persistent D-dimer elevation is recognized as a poor prognostic sign in DIC. Kudo et al. noted that sustained D-dimer elevation signals ongoing coagulation activation (21). Our study is among the first to quantify this persistence in APL-related ICH. The combined model outperformed either marker alone. This suggests that fibrinogen depletion and fibrinolytic activation, though linked, contribute non-redundant information. The correlation between the two rates was substantial (r = 0.72), reflecting their shared dependence on the underlying fibrinolytic process.

Platelet trajectories did not differ between groups. Both cohorts showed steady platelet recovery. Ten et al. reported that thrombocytopenia contributes to bleeding risk in APL (22). Rigouzzo et al. argued that hypofibrinogenemia is the dominant driver of hemorrhage (23). Our findings support the latter view. Platelet counts rose similarly, yet expansion occurred in the setting of persistent fibrinolytic activity. This dissociation suggests that platelet transfusion alone may be insufficient when fibrinolysis is uncontrolled. Chu et al. recommend maintaining fibrinogen above 1.6 g/L to prevent bleeding (16). These are static targets. Our data suggest that a patient meeting these targets may still be at risk if the fibrinogen trajectory is downward. Conversely, a patient below target but with a rising fibrinogen level may be stabilizing. The decline rate adds temporal context to absolute thresholds.

The fibrinogen decline rate remained independently associated with expansion after multivariable adjustment. Established predictors such as initial hematoma volume and midline shift were included. In spontaneous ICH, the CTA spot sign predicts expansion with an AUC ranging from 0.75 to 0.85. The BRAIN score incorporates clinical and imaging variables to estimate expansion risk. Neither tool was developed for coagulopathic ICH. Our combined model achieved an AUC of 0.94. Direct comparison with the spot sign is not possible in this retrospective study, as CTA was not routinely performed. Nevertheless, the discriminative performance appears favorable. The coagulation-based approach may complement imaging-based prediction. Future studies should evaluate whether combining dynamic coagulation markers with the spot sign improves risk stratification further.

Thirty-day mortality was substantially higher in the expansion group. The neurosurgical intervention rate did not differ between groups. This observation is consistent with reports by Chu et al., who documented that early hemorrhagic death in APL often occurs before surgical intervention can be attempted (16). Expansion may reflect a systemic coagulopathy rather than a localized surgical problem. Our data do not resolve whether expansion is a mediator or a marker of mortality. Regardless, the decline rate identifies a subgroup with a 50% mortality risk. This prognostic information is clinically actionable. It may guide goals-of-care discussions and resource allocation in the intensive care unit.

The clinical applicability of the fibrinogen decline rate deserves emphasis. The proposed cut-off of 0.35 g/L/day corresponds to a drop of approximately 0.35 g/L within the first 24 h. This is readily detectable with twice-daily coagulation monitoring, which is standard practice in most leukemia centers. The negative predictive value was 88.1%. A decline rate below this threshold may be used to rule out subsequent expansion. This high negative predictive value makes the marker suitable for identifying low-risk patients who may be managed conservatively. The positive predictive value was 65.4%. Not all patients with rapid decline develop expansion. This moderate positive predictive value indicates that the decline rate should be used for screening rather than definitive prediction. Combining it with imaging markers may improve specificity.

The D-dimer increase rate showed similar discriminative ability in univariate analysis. Its AUC of 0.88 was comparable to the fibrinogen decline rate. However, it lost significance after adjustment. This likely reflects collinearity. Both markers capture the hyperfibrinolytic state. The combined model incorporating both rates achieved the highest AUC. From a practical standpoint, fibrinogen is more readily available in real time than D-dimer in many centers. Fibrinogen can be measured rapidly with point-of-care devices. The decline rate can thus be calculated at the bedside without delay. D-dimer, while valuable for research and risk modeling, may be less practical for minute-to-minute clinical decisions (24).

Our findings should be interpreted in the context of several limitations. First, the retrospective single-center design carries inherent selection bias. Second, the sample size, though adequate for the events-per-variable ratio of the primary models, limits the precision of the combined model; we therefore treated the seven-predictor model as exploratory and fitted it with Firth's penalized regression. External validation in multicenter cohorts is essential before clinical adoption. Third, the ABC/2 method for hematoma volumetry has known inaccuracies for irregular hemorrhages. Two independent raters performed measurements to mitigate this. Fourth, patients who died or were transferred before follow-up CT were excluded. This introduces survivorship bias. The sickest patients with the most rapid fibrinogen decline and fatal expansion were likely excluded. Our model therefore applies primarily to patients surviving the initial hours who are candidates for aggressive intervention. The true association between decline rate and expansion may be stronger than we observed. Fifth, transfusion protocols were not standardized across the eight-year study period. Clinicians may have transfused higher-risk patients more aggressively. This would bias results toward the null. The persistent association despite this potential bias strengthens confidence in the biological signal. Sixth, we did not measure specific fibrinolysis markers such as plasmin-antiplasmin complex or plasminogen activator inhibitor-1. D-dimer served as a surrogate. Future studies incorporating these markers could refine the mechanistic understanding. Seventh, subgroup analyses by ATRA timing or surgical approach were not feasible due to sample size constraints.

## 5 Conclusion

The fibrinogen decline rate is a novel independent predictor of hematoma expansion in APL-related ICH, independent of the baseline fibrinogen level. The concept of dynamic coagulation monitoring extends the current paradigm of static risk assessment. The proposed cut-off is simple, clinically accessible, and has high negative predictive value. Prospective validation in multicenter cohorts is warranted. If confirmed, real-time fibrinogen trajectory monitoring could become a standard component of early risk stratification in this high-risk population.

## Data Availability

The raw data supporting the conclusions of this article will be made available by the authors, without undue reservation.
